# Linker histone variant H1t is closely associated with repressed repeat-element chromatin domains in pachytene spermatocytes

**DOI:** 10.1186/s13072-020-00335-x

**Published:** 2020-03-04

**Authors:** Iyer Aditya Mahadevan, Sanjeev Kumar, Manchanahalli R. Satyanarayana Rao

**Affiliations:** 1 Molecular Biology and Genetics Unit, Jawaharlal Nehru Centre for Advanced Scientific Research, Bangalore, India; 2 BioCOS Life Sciences Private Limited, SAAMI Building, 851/A, AECS Layout, B-Block, Singasandra Hosur Road, Bangalore, India

**Keywords:** Linker histones, H1t, LTR, LINE, Histone variants, Mammalian spermatogenesis

## Abstract

**Background:**

H1t is the major linker histone variant in pachytene spermatocytes, where it constitutes 50–60% of total H1. This linker histone variant was previously reported to localize in the nucleolar rDNA element in mouse spermatocytes. Our main aim was to determine the extra-nucleolar localization of this linker histone variant in pachytene spermatocytes.

**Results:**

We generated H1t-specific antibodies in rabbits and validated its specificity by multiple assays like ELISA, western blot, etc. Genome-wide occupancy studies, as determined by ChIP-sequencing in P20 mouse testicular cells revealed that H1t did not closely associate with active gene promoters and open chromatin regions. Annotation of H1t-bound genomic regions revealed that H1t is depleted from DSB hotspots and TSS, but are predominantly associated with retrotransposable repeat elements like LINE and LTR in pachytene spermatocytes. These chromatin domains are repressed based on co-association of H1t observed with methylated CpGs and repressive histone marks like H3K9me3 and H4K20me3 in vivo. Mass spectrometric analysis of proteins associated with H1t-containing oligonucleosomes identified piRNA–PIWI pathway proteins, repeat repression-associated proteins and heterochromatin proteins confirming the association with repressed repeat-element genomic regions. We validated the interaction of key proteins with H1t-containing oligonucleosomes by use of ChIP-western blot assays. On the other hand, we observe majority of H1t peaks to be associated with the intergenic spacer of the rDNA element, also in association with SINE elements of the rDNA element. Thus, we have identified the genomic and chromatin features of both nucleolar and extranucleolar localization patterns of linker histone H1t in the context of pachytene spermatocytes.

**Conclusions:**

H1t-containing repeat-element LINE and LTR chromatin domains are associated with repressive marks like methylated CpGs, histone modifications H3K9me3 and H4K20me3, and heterochromatin proteins like HP1β, Trim28, PIWIL1, etc. Apart from localization of H1t at the rDNA element, we demonstrate the extranucleolar association of this linker histone variant at repeat-associated chromatin domains in pachytene spermatocytes. We hypothesize that H1t might induce local chromatin relaxation to recruit heterochromatin and repeat repression-associated protein factors necessary for TE (transposable element) repression, the final biological effect being formation of closed chromatin repressed structures.

## Background

The chromatosome is a structural unit of chromatin, consisting of about 166 bp DNA wrapped around the histone octamer with histone H1 [1, 2]. Despite their significant roles in various chromatin-templated biological events, linker histones are not studied in great detail as core histones. H1s possess a unique structure namely, the N-terminal domain, conserved trypsin-resistant globular domain, and C-terminal domain [3, 4]. The N and C-terminal domains of H1 are divergent and largely unstructured in solution [5–7]. Its globular domain is the nucleosome-binding domain that protects a 20-bp of nucleosomal DNA, just like the full-length H1. On the other hand, the C-terminal domain of H1 is the primary determinant of DNA binding in cells [8]. Along with core histones, the linker histone (H1) is one of the five major histone families associated with the eukaryotic chromatin. In mice and humans, 11 H1 variants have been identified to date that includes seven somatic subtypes (H1.0, H1.1–H1.5, and H1x), three testis-specific variants (H1t, HILS1, and H1T2) and one oocyte-specific variant (H1oo).

Mammalian spermatogenesis is an excellent model system to study the biological roles of linker histone variants, as various germ cell variants like H1t, HILS1 and H1T2 are expressed in a stage-specific manner. The testicular linker histone variant H1t is expressed from preleptotene spermatocytes till early round spermatids (in mouse) or till late-round spermatids (in humans) [9–12]. Even though H1t mRNA is detected in spermatogonia, the protein is absent. H1t accounts for about 50–60% of total H1 in these cell types [13–15]. Surprisingly, the loss of H1t has been shown to cause no detectable defects during spermatogenesis in mice. There are conflicting reports on the phenotypes observed in H1t null mice. For example, H1 subtypes are deposited and been reported to compensate for the loss of H1t [16]. In other reports, H1t-deficient chromatin is shown to be H1 free [17, 18]. Biophysically, H1t is a poor condenser of chromatin in comparison to somatic H1 subtype H1.d (H1.3) as demonstrated by in vitro CD (circular dichroism) spectroscopic studies [19, 20]. This property was attributed to a lack of DNA-binding motifs like SPKK in the C-terminal domain of H1t [21–24]. SPKK and other motifs are responsible for DNA condensation property in histone H1d. Also, in comparison with somatic H1s, a K52Q substitution in the globular domain causes reduction in DNA-binding affinity of H1t [25].

Various defense mechanisms have evolved in germ cells of different species to prevent expression of the retrotransposable elements, thus limiting their mutagenic potential. DNA methylation at LINE and LTR retrotransposable elements can be dependent on piRNA expression or not, also termed as piRNA-dependent and piRNA-independent, respectively [26]. DNA methylation at LINE retrotransposons are piRNA-dependent [26]. In contrast, silencing of LTR retrotransposons by DNA methylation can be piRNA-dependent or independent, with DNA methylation at most of the LTRs being piRNA-independent. Another set of transposable elements like SINE does not exhibit piRNA-dependent repression [27]. This suggests that retrotransposon inactivation in germ cells is more complex and their mechanism by the action of important players is currently being investigated in great detail.

Retrotranscripts originating from TE (transposable element) sequences in the nucleus are cleaved into sense and antisense piRNAs. The sense RNAs associate with GasZ-containing MILI–TDRD1 complexes within pi-bodies [28]. On the other hand, the antisense RNAs associate with MIWI2–TDRD9 complexes and then localize to MAEL-containing piP bodies [29, 30]. Degradation of retrotranscripts occurs by the exchange of sense and antisense transcripts in the cytoplasmic compartments. MIWI2–TDRD9 complexes can also induce feedback signaling into the nucleus to facilitate recruitment of Dnmt3L/3A machinery to induce de novo DNA methylation at the target TE loci. Some of the essential proteins like MIWI, MAEL, GasZ related to TE repression are also expressed during later stages of spermatogenesis. Mutations in these genes result in pachytene arrest and male infertility.

Our present main aim was to determine the genome-wide occupancy of linker histone variant H1t in pachytene spermatocytes, which would give an idea about its association with specific chromatin domains and consequent biological functions. H1t is not exclusively expressed in testis, but also expressed in various cancer cells and mouse embryonic stem cells [31]. Recently, H1t-ChIP-sequencing was carried out in human cancer cell lines and mouse ESCs. H1t was found to be majorly associated with the rDNA element of the nucleolus in these cells [31]. Also, in the same study, various extranucleolar foci of H1t were observed in spermatocytes, which provided major inspiration for the present study to characterize the extranucleolar localization of the linker histone variant H1t in mammalian spermatocytes. It is also important to characterize the binding sites of H1t in the pachytene genome, as H1t is the dominant H1 in these cells, constituting about 50–60% of total H1 content [13–15]. This information along with proteins associated with these chromatin domains would give clues towards biological significance of histone H1t in male germ cells due to which they have majorly replaced somatic H1s in the germ cells. We demonstrate that H1t is associated with LTR and LINE repeat-element chromatin domains in pachytene spermatocytes. H1t is also closely associated with histone marks H3K9me3 and H4K20me3 and PIWI–piRNA pathway-related proteins suggesting that these chromatin domains represent repressed chromatin domains in vivo.

## Results

### Validation of specificity of H1t antibodies

**Fig. 1:**
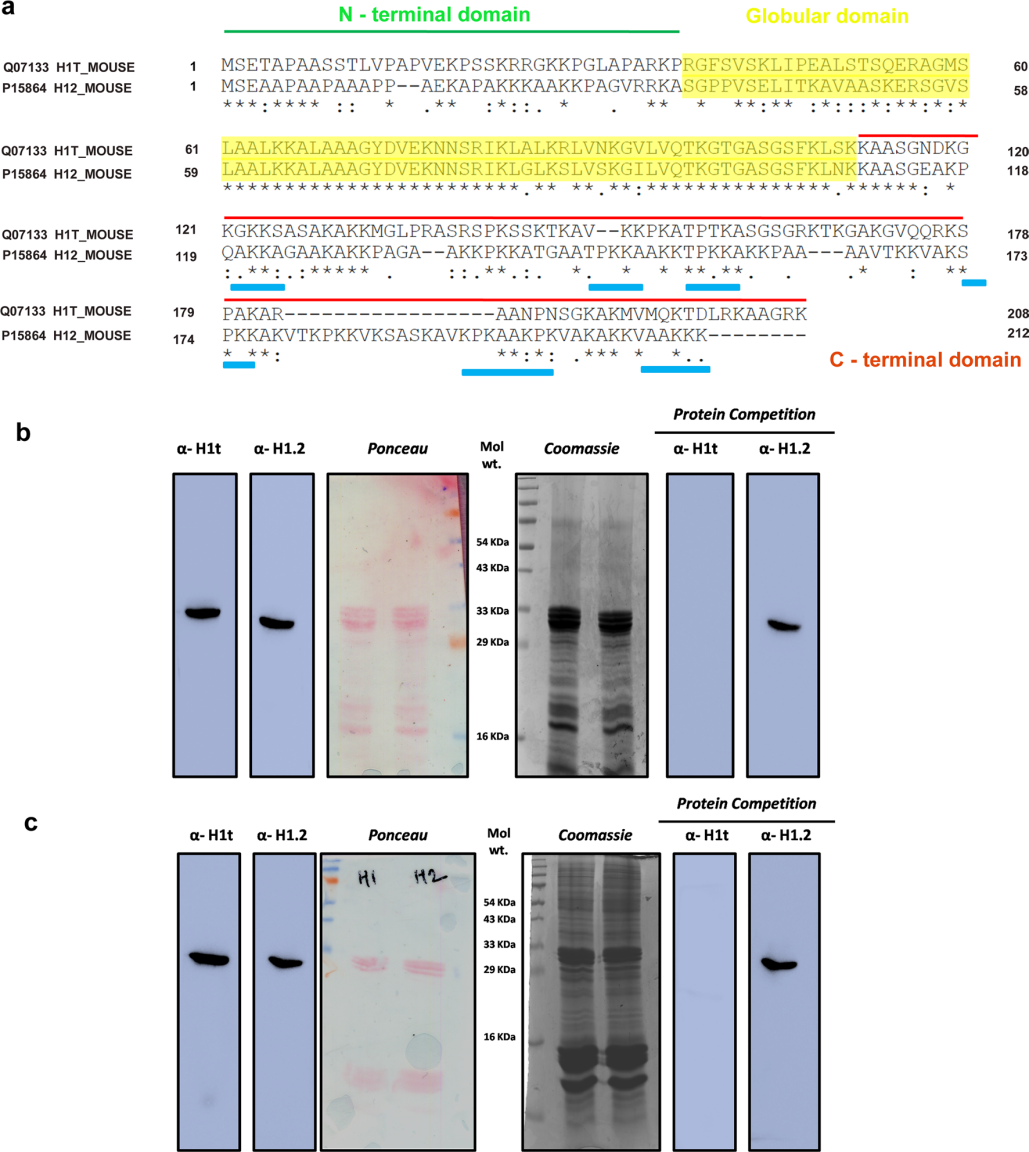
Generation and validation of specificity of H1t-specific antibodies. **a** The tripartite structure of linker histones—the linker histones possess three major domains: N-terminal domain, globular domain, and the C-terminal domain. The DNA-binding motifs like SPKK that are present in the somatic linker histone H1.2 have been underlined using blue lines. Since the C-terminal of H1t protein is highly divergent in comparison with other variants, we used 112–207 amino acid residues as protein fragment for the generation of H1t-specific antibodies in rabbits. **b** Western blotting of H1t and H1.2 antibodies against perchloric acid extracts prepared from P20 mouse testicular cells. The anti-H1t and anti-H1.2 antibodies showed reactivity to the specific linker histones, as seen in the blot images on the left, where bands corresponding to molecular weights of H1t and H1.2 were obtained. The blots on the right indicate the immunoblotting performed with the H1t and H1.2 antibodies after preincubation with the recombinant H1t C-terminal protein fragment, before their addition to the blot. Ponceau and Coomassie-stained images are given for reference. **c** Immunoblotting using H1t and H1.2 antibodies against 0.4 N H_2_SO_4_ acid extracted histones prepared from P20 mouse testicular cells. The blots on the left show the western blotting data performed using H1t and H1.2 antibodies against the acid extracted histones. The blots on the right indicate the western blotting data performed using H1t and H1.2 after preincubation with the recombinant H1t C-terminal protein fragment. Ponceau and Coomassie-stained images are given for reference

As mentioned earlier, our main aim was to determine the genome-wide localization of linker histone variant in pachytene spermatocytes, for which we required to generate highly specific H1t antibodies. The comparison of protein sequences of H1t with somatic linker histone variant H1.2 is given in Fig. 1a. As explained earlier, H1t lacks DNA-binding motifs that are present in the somatic linker histones (Fig. 1a, blue lines). To address the biological functions, we first generated H1t-specific antibodies in rabbits. Since C-terminal domain is highly divergent between H1t and somatic H1s, we cloned and purified the C-terminal protein fragment of H1t (Additional file 1: Figure S1A), then used as an antigen to generate polyclonal antibodies in rabbits. We determined the specificity of the antibodies by ELISA, western blotting and protein competition assays. By ELISA assays, we found that the sera, as well as purified antibody, reacted with the recombinant H1t C-terminal protein fragment (Additional file 1: Figure S1B and Additional file 1: Figure S1C, respectively). Western blotting also showed specific reactivity with the protein corresponding to H1t in the perchloric acid testicular extracts (Fig. 1b, α-H1t lane). Also, we observe a different band when H1.2 antibodies were used for immunoblotting (Fig. 1b, α-H1.2 lane), suggesting that the molecular weights of the bands were specific to these variants and the antibodies did not cross-react with other variants. Additionally, we observed, after preincubation with the H1t C-terminal antigen, the reactivity of H1t antibodies were abolished, thereby suggesting the specificity towards the H1t linker histone variant (Fig. 1b, protein competition, α-H1t lane). However, as can be seen in Fig. 1b (protein competition, α-H1.2 lane) the reaction to H1.2 was not competed out with the H1t C-terminal antigen. We obtained similar results with immunoblotting using H1t and H1.2 antibodies against acid extracts (0.4 N H_2_SO_4_) derived from P20 mouse testicular cells, wherein we observed the specific reactivity towards the variant histones (Fig. 1c, α-H1t and α-H1.2 lanes). The C-terminal antigen blocked the specific reactivity of H1t antibodies (Fig. 1c, protein competition, α-H1t lane) but not the H1.2 antibodies (Fig. 1c protein competition, α-H1t lane) against acid extracted nuclear proteins. The H1t antibodies did not react with the somatic H1-containing liver histone extracts as demonstrated by immunoblotting assays (Additional file 1: Figure S1D).

All the immunoblotting results observed in mouse testicular cells were also found to be true in rat testicular extracts. We observed specific reactivity of H1t antibodies against perchloric acid extracts and acid extracted histones, both extracted from rat testicular cells and observed the specific band corresponding to H1t by western blotting (Additional file 2: Figure S2A and Additional file 2: Figure S2B, α-H1t lanes). Further, the preincubation of H1t antibodies with the C-terminal antigen abolished the reactivity towards the H1t of the linker histones and acid soluble nuclear extracts (Additional file 2: Figure S2A and B, protein competition, α-H1t lanes). These results were additionally confirmed by mass spectrometry, as H1t was the only variant to be associated with H1t-containing oligonucleosomes (see later). We further determined the immunostaining pattern of H1t across the various stages of meiotic prophase I. We observed uniform distribution of H1t protein across the leptotene, zygotene and pachytene cells (Additional file 3: Figure S3A), consistent with being the dominant H1 in spermatocytes. The combination of western blotting, ELISA and mass spectrometry assays thus establish the specificity of the in-house-generated H1t antibodies.

### Genome-wide occupancy of linker histone variant H1t in pachytene spermatocytes

**Fig. 2:**
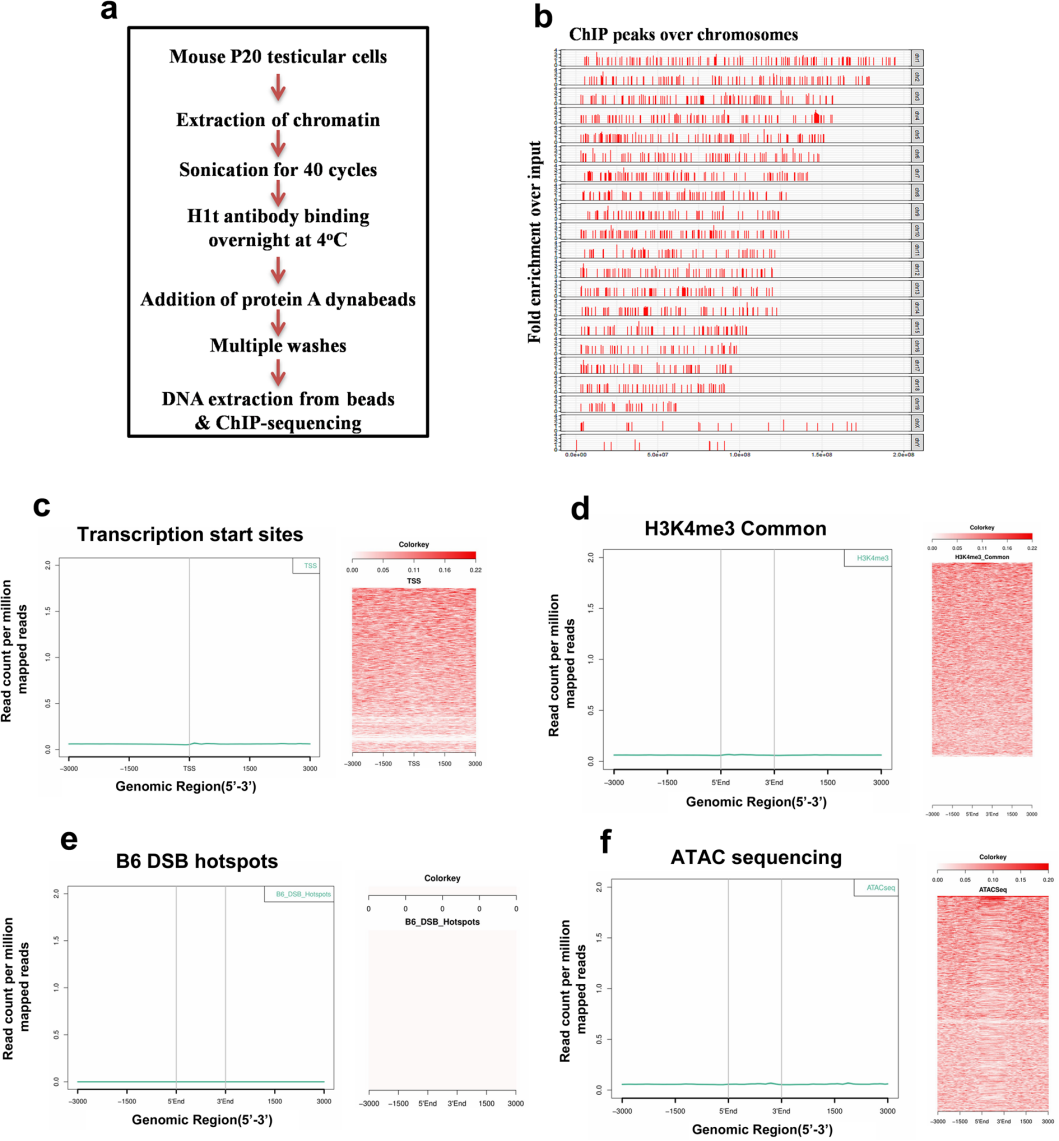
Localization of linker histone variant H1t at TSS, active gene promoters, recombination hotspots and open chromatin regions (ATAC-seq-positive regions). **a** Workflow of the experimental protocol used for the H1t ChIP-sequencing technique. **b** Chromosome-wide distribution of H1t peaks in the pachytene genome. The *y*-axis represents the fold enrichment observed for H1t IP over background input, while the *x*-axis represent the various H1t-occupied locations along the length of each chromosome. The normalization of high fold enriched peaks has been done to 3.5-fold change and the lower threshold has been maintained for fold change = 2. Analysis of overlap between H1t and **c** TSS of the mouse (GENCODE), **d** H3K4me3 common representing the TSS-associated H3K4me3 marks (GSE93955), **e** DSB hotspots (GSE93955), **f** open chromatin regions of pachytene spermatocytes [35]. H1t were observed to be not enriched at active gene promoters, TSS regions, and open chromatin genomic regions. Information in **c**–**f** represent the overlap that has been determined using aggregation plots (left panels) and heat maps (right panels)

Since H1t is a linker histone and a component of chromatin, we carried out ChIP-sequencing with the crosslinked chromatin to determine the occupancy sites of H1t in the pachytene chromatin of mouse. The experimental workflow of the ChIP protocol is given in Fig. 2a. The profile of DNA fragments obtained after various cycles of sonication is given in Additional file 3: Figure S3B. We chose mouse testis as the model system for the study, since various datasets related to biological processes like meiotic recombination, transcription have been well characterized in the mouse species.

By ChIP-sequencing and the related data analysis, we obtained statistically significant (p value <= 0.05) 48,681 peaks of H1t occupancy (refer methods section for details). The chromosome-wide distribution of these H1t peaks is shown in Fig. 2b. As can be seen in Fig. 2b, linker histone variant H1t is depleted from the XY body relative to the autosomes (224 peaks in chromosome X and 89 peaks in chromosome Y). This corroborates with previously published data wherein H1t was found to be depleted from the XY body and is associated with autosomal chromatin domains in pachytene spermatocytes [32], highlighting the robustness of our ChIP-sequencing data. To reiterate, linker histones are known to be generally depleted from active TSS, open chromatin structures in vivo, except some variants like H1x, H1.1 [33]. We wanted to determine whether the variant H1t is associated with genomic regions related to transcription, meiotic recombination, etc. We performed overlap analysis of H1t genomic peaks with other ChIP-sequencing datasets and the results are represented as aggregation plots and heat maps. Both these methods show the spatial distribution of reads within target genomic regions [34]. Aggregation plots as given in Fig. 2c, d (left panels), shows that H1t is not significantly enriched at transcription start sites as well as H3K4me3-marked active gene promoters. The heat maps corroborate with this data as no significant enrichment was observed for H1t reads at TSS and active gene promoters (Fig. 2c, d, right panels). When analyzed with DSB hotspots, we observe H1t also not to be closely associated with recombination hotspot genomic sequences (Fig. 2e). Furthermore, when performing the overlap analysis of H1t ChIP-seq dataset with ATAC-sequencing dataset available for pachytene spermatocytes [35], we observed that H1t is not majorly associated with open chromatin regions (ATAC-seq-positive genomic regions) in the pachytene genome (Fig. 2f). To confirm this further, we went ahead by performing forward and reciprocal immunoprecipitation assays to determine whether active chromatin domains bearing H3K4me3 histone mark is associated with H1t linker histone variant in testicular cells. We observe H1t to be not associated with H3K4me3-containing oligonucleosomes (Additional file 3: Figure S3C). Also, H3K4me3 histone mark is not associated with H1t-positive oligonucleosomes (Additional file 3: Figure S3D). Apart from the aggregation plots and biochemical assays, we also observe lower percentages of H1t peaks overlapping with TSS, DSB hotspots, active gene promoters and open chromatin regions (Additional file 4: Figure S4A). These observations suggested to us that H1t might be associated with chromatin regions whose functional consequence would be to ultimately form condensed chromatin structures in vivo.

**Fig. 3:**
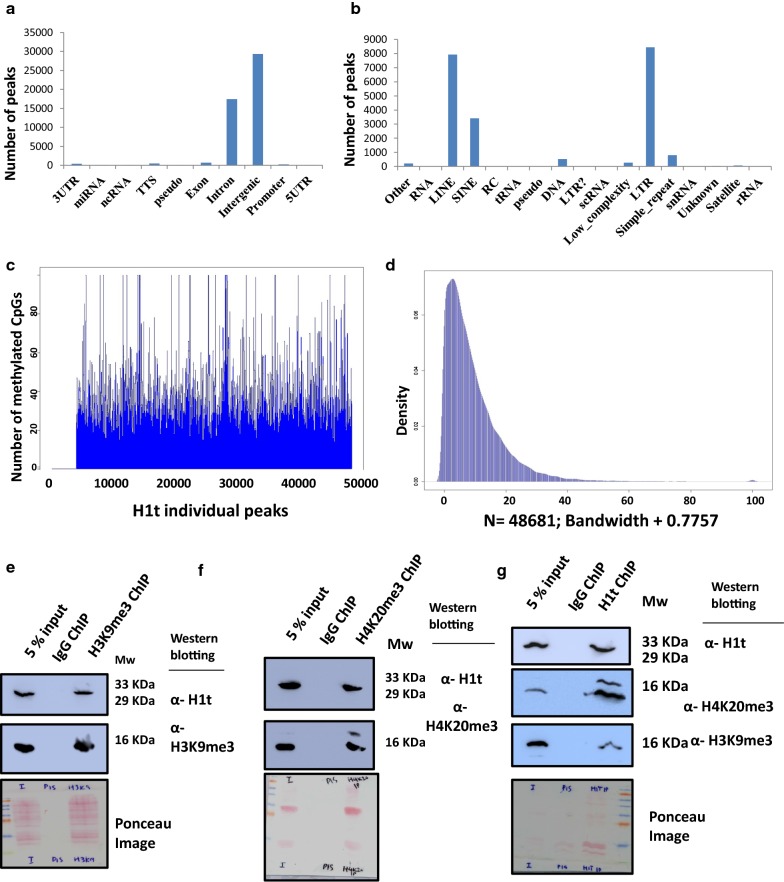
Localization of linker histone H1t at CpG-methylated repeat element chromatin domains. **a** Annotation of H1t-bound genomic regions using HOMER. **b** Annotation of H1t peaks at repeat elements showing its predominant association with LINE and LTR subclasses of retrotransposable elements. **c** Profile of methylated cytosines (SRS557654) across all the H1t peaks. More than 90% of the H1t peaks overlap with methylated CpGs. The y-axis is the count of methylated CpG positions at each peak. Some peaks with more than 100 methylated cytosines have been truncated to 100 for better visualization of the overall plot. **d** Density plot of H1t peaks overlapping with methylated CpGs (SRS557654). The y-axis represents the density function and the x-axis represents the bandwidth parameter. N represents the number of observations. Co-immunoprecipitation assays showing the coexistence of H1t-containing oligonucleosomes with histone marks H3K9me3 and H4K20me3. **e** H3K9me3-ChIP showing the co-association with linker histone variant H1t in testicular chromatin. **f** Linker histone variant H1t is associated with H4K20me3-ChIP elute fraction. **g** Reciprocal immunoprecipitation assays showing that H1t-positive chromatin fragments to be associated with histone marks H3K9me3 and H4K20me3 in vivo. Information in **e**–**g**. The first lane is the input fraction; the second lane is the IP using the non-specific IgG isotype control, the third lane is the IP with the mentioned antibodies (anti-anti-H3K9me3/anti-H4K20me3/anti-H1t). The antibodies labeled alongside the blot refer to the antibodies used for western blotting. Ponceau-stained blots are given for reference

Since H1t is not significantly enriched in TSS, active gene promoters, DSB hotspots, and ATAC-seq positive regions, the primary question remained to what genomic regions are H1t localized at. Upon initial annotation of the H1t-occupied genomic regions, we observed that H1t is majorly associated with intergenic and intronic regions (Fig. 3a). On further annotation of the H1t-bound genomic regions, we observed that the majority of the H1t were closely associated with LINE and LTR classes of repetitive elements, and SINE to a lesser extent (Fig. 3b). We therefore conclude that H1t is localized to retrotransposable elements LINE, LTR and SINE in vivo. Even though H1t is depleted from the XY body [32], the localization of the few peaks of H1t that we have observed in the chromosomes X and Y correlates with the repeat elements (specially intergenic, LTR and LINE elements). In the chromosome X, H1t is predominantly localized to LTR (99 peaks), LINE (56 peaks), SINE (6 peaks), intergenic (61 peaks) genomic regions out of the total 224 peaks. In chromosome Y (total 89 peaks), H1t is localized predominantly at intergenic (39 peaks), LINE (16 peaks) and LTR (39 peaks) repeat elements. It is now well established that these retrotransposable elements are repressed in the germ cells by the action of RNA interference machinery and small RNAs. Despite millions of years of divergence, the machinery involving RNAi machinery and piRNAs in preventing transposable element (TE) expression have been conserved in fungi, plants, and animals. piRNAs are essential for de novo DNA methylation at these TE loci crucial for silencing of LINE and LTR elements in embryonic male germ cells. DNA methylation is carried out by the methyltransferase enzyme Dnmt3A in early germ cells, the loss of which results in male infertility due to meiotic failure [36]. Therefore, DNA methylation of repeat elements is a major mechanism for preventing TE expression and is critical for success of productive spermatogenesis. Since the piRNA pathway machinery repress TE by DNA methylation, our next question was to determine whether H1t-associated genomic regions are also associated with methylated genomic sequences. We carried out the overlap analysis of our H1t ChIP-sequencing data with the already published bisulfite sequencing dataset available for P20 mouse testicular cells [37]. As can be seen in Fig. 3c, d, we observed that more than 90% (44,720 peaks) of H1t peaks were associated with methylated CpGs at these repetitive elements. These observations suggested that H1t, apart from its major association with classes of repetitive elements LINE and LTR elements, are methylated in pachytene-enriched P20 mouse testicular cells.

In addition to DNA methylation, repressive histone modifications like H3K9me3 [38] and H4K20me3 [39] facilitate silencing of the retrotransposable elements. H3K9me3 histone mark is present on LINE and LTR repeat elements in germ cells and is dependent on the function of piRNA pathway [38]. H3K9 methylation is an important epigenetic mark in transcriptional silencing and heterochromatin formation. H3K9me3 formation is mediated by histone methyltransferases (HMTs) Suv39h1 and Suv39h2 in germ cells [40–42]. We went ahead to determine whether H1t-containing oligonucleosomes are associated with repressive histone marks like H3K9me3 and H4K20me3 in vivo. By employing oligonucleosome IP assays, we found that H3K9me3 and H4K20me3 did pull down H1t protein (Fig. 3e, f, respectively). Also, by reciprocal IP assays, we observed H1t-associated oligonucleosomes to be positive for H3K9me3 and H4K20me3 histone marks in vivo (Fig. 3g). These biochemical assays demonstrate the association of repressive histone marks H3K9me3 and H4K20me3 with H1t-bound chromatin fragments in pachytene-enriched P20 testicular cells. Thus, we provide strong evidence to the fact that H1t-containing genomic regions are associated with DNA methylation and repressive histone modifications H3K9me3 and H4K20me3 in P20 mouse testicular cells that are enriched in pachytene spermatocytes.

### Localization of linker histone variant H1t in the rDNA element of the pachytene spermatocyte

**Fig. 4:**
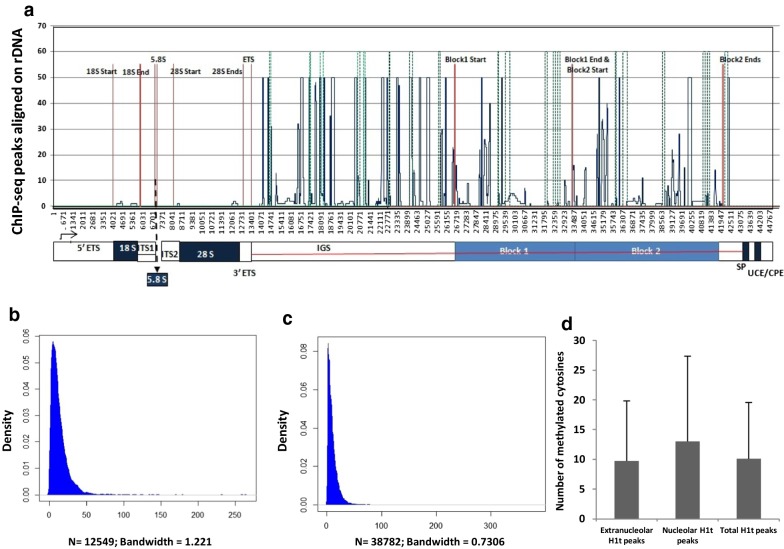
Localization of linker histone H1t in the mouse rDNA element. **a** Peak distribution of H1t across various regions of the rDNA element [43]. Distribution of H1t peaks with respect to SINE elements (green dotted lines) localized in the rDNA element. The red lines demarcate various regions of the rDNA element, blue lines are the H1t peaks, and the various regions of the rDNA element have been labeled below the peak distribution maps. Density plot of H1t peaks overlapping with methylated CpGs (SRS557654) in **b**. rDNA and **c** extranucleolar genomic regions. The y-axis represents the density function and the x-axis represents the bandwidth parameter. N represents the number of observations. The red lines demarcate various regions of the rDNA element [43], blue lines are the H1t peaks, and the various regions of the rDNA element have been labeled below the peak distribution maps. **d** Level of DNA methylation in the extranucleolar, nucleolar and the total H1t peaks. The data have been plotted in terms of the number of methylated cytosines as mean ± SD

Linker histone variant H1t was previously demonstrated to be localized to the rDNA element of the nucleoli in spermatocytes by immunofluorescence assays [31]. We confirmed the association of H1t in the rDNA element of pachytene spermatocytes, wherein we observe 13,008 peaks to be localized at the known mouse rDNA element. Interestingly, the majority of the H1t peaks are localized in the intergenic spacer of the rDNA element (Fig. 4a).

The rDNA element harbors many repetitive elements, the dominant element being SINE [43]. SINE elements constitute about 20% of the total rDNA element in the mouse. We observe that H1t peaks are located at or close to the vicinity of the predominant SINE elements (marked in green dotted lines) in the rDNA element of the pachytene spermatocyte (Fig. 4a). We also wondered whether the H1t peaks localized at the repetitive elements of the rDNA element are associated with DNA methylation. True to our intuition, we observed more than 95% (12,550 peaks) of H1t peaks are indeed associated with methylated CpGs at the rDNA element (Fig. 4b and Additional file 4: Figure S4B). In addition to the extranucleolar localization of H1t at the retrotransposon classes LINE and LTR (Fig. 4c), we also observe significant overlap of H1t at the repressed repetitive elements of the rDNA element, characterized by occupancy of methylated CpGs. A detailed comparison of association of DNA methylation-associated H1t peaks with nucleolar and extranucleolar chromatin domains are given in Additional file 5: Fig. 5a, b. We also observe an increased status of CpG methylation in the H1t-bound rDNA genomic regions compared to the H1t-bound extranucleolar peaks (Fig. 4d). Thus, in both the nucleolar and extranucleolar mouse genome of P20 testicular cells, linker histone variant H1t occupy methylated CpG repeat-associated chromatin domains.

**Fig. 5:**
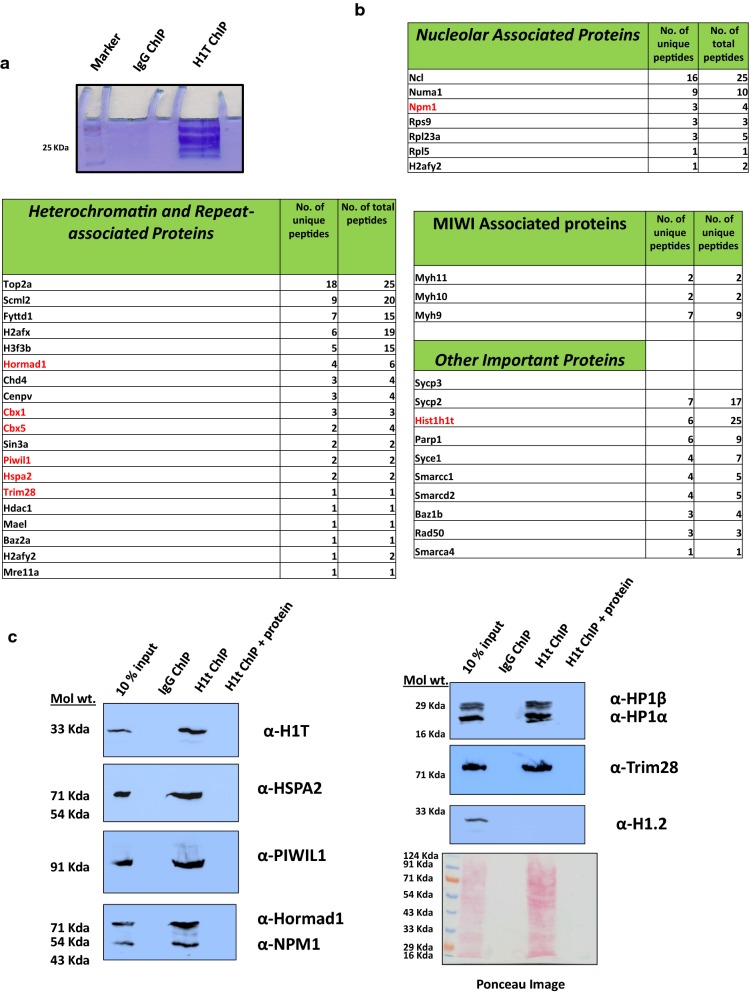
List of key proteins associated with H1t-positive chromatin fragments in pachytene spermatocytes as determined by mass spectrometry. **a** Coomassie-stained gel showing the success of the H1t-ChIP technique. The first lane represents the protein marker, the second lane is the ChIP carried out using the rabbit preimmune IgG antibodies and the third lane is the ChIP carried out using H1t antibodies. **b** The important proteins that are associated with H1t-containing oligo nucleosomes can be broadly divided into four major classes: nucleolar function, heterochromatin and repeat-associated proteins, MIWI-associated proteins and other important proteins. The proteins indicated in red color have been selected for further validation by co-IP assays. **c** Validation of H1t-associated proteins by ChIP-western blotting technique. To validate further the association of key proteins with H1t-associated chromatin, we carried out western blot analysis of the H1t-positive oligo nucleosomes. We observe proteins related to the nucleolus (NPM1), heterochromatin related (Hormad1, Trim28, HP1β, HP1γ), PIWI pathway-related (PIWIL1, Hspa2) are associated with H1t-containing ChIP fraction. The first lane in all the blots represents the 10% input fraction, second lane ChIP with the non-specific isotype control, and the third lane ChIP with the H1t antibody, and the fourth lane is the ChIP carried out along with addition of C-terminal H1t recombinant protein fragment (10 µg). The antibodies labeled in alpha alongside the blot represent the antibodies used for western blotting

### Mass spectrometric identification of proteins associated with H1t-bound chromatin fragments

H1t being the major linker histone component of pachytene chromatin, we wanted to identify the proteins that co-associate with H1t-containing chromatin fragments. For this purpose, after carrying out the H1t ChIP, we performed mass spectrometric analysis of the eluted proteins, to identify the H1t-associated proteins in the chromatin context. The proteins were identified based on the enrichment of proteins observed in the H1t ChIP fraction compared to non-specific rabbit IgG ChIP fraction (Fig. 5a). Interestingly, we observed H1t be the only linker histone variant associated with the H1t ChIP fraction. This provides additional strength for our studies regarding the specificity of the H1t antibody, highlighting the robustness of our observations.

Further, we observed that the H1t-associated proteins could be identified into three classes—nucleolar-associated, repeat element and heterochromatin-associated and other important proteins (Fig. 5b). We expected association of nucleolus related proteins as H1t is shown to be localized at the rDNA element of mouse spermatocytes (Fig. 5b, nucleolar proteins) [31]. Importantly, we observed various PIWI–piRNA pathway proteins such as Piwil1(MIWI) and its associated proteins (Myh9, Myh10, Myh11), HSPA2, MAEL to be associated with H1t-oligonucleosomes (Fig. 5b, repeat-associated and heterochromatin proteins, MIWI-associated proteins). Piwil1 protein is a bonafide slicer (small-RNA-directed endonuclease) in postnatal germ cells wherein the loss of Piwil1 has been shown to cause male fertility due to the upregulation of LINE1 retrotransposon transcripts [44]. Also, Tdkrh1 is known to interact with PIWI proteins and is an essential factor implicated in piRNA biogenesis [45]. These results provide additional evidence to the association of H1t with proteins related to TE silencing in pachytene spermatocytes. The entire list of proteins obtained after mass spectrometry is given in Additional file 8. Also, the list obtained on comparison of H1t-associated proteins with the comprehensive published list of heterochromatin proteins [46] is given in Additional file 9. Further, we have validated the association of H1t with some of the key proteins by carrying out ChIP assays followed by western blotting (using the antibodies for detection of proteins highlighted in red), and observed that H1t is associated with proteins related to nucleolar function (Npm1), repeat-repression and heterochromatinization (HP1β, HP1α, Piwil1, Hspa2, Hormad1, Trim28) in vivo (Fig. 5c, third lanes). H3K9me3-positive nucleosomes are the docking sites for the binding of chromodomain-containing HP1 proteins establishing the chromatin condensed structure. This chromatin template has been proven for transcriptional repression, also leading to spreading and amplification of heterochromatin domain. The fact that H1t-containing nucleosomes associate with H3K9me3, HP1α, HP1β gives a strong indication for the localization of linker histone H1t at repressed repeat-element chromatin domains.

In addition to H3K9me3 and H4K20me3, there could be other histone marks that could be signatures of repeat-element nucleosomes. Uhrf1 has been shown to interact with PRMT5 (arginine methyltransferase) to catalyze the formation of H4R3me2s and H3R2me2s. These marks also act together with PIWI proteins to facilitate retrotransposon silencing in the germline [47]. We found that H1t-oligonucleosomes associate with Tdkrh, a known interacting protein partner with Uhrf1 and PIWI proteins, further supporting our observations of association of H1t with repressed-repeat element chromatin domains in pachytene spermatocytes. Importantly, preincubation of the H1t-antibodies with C-terminal protein of H1t blocked the reactivity of the antibodies, reconfirming the validity of our observations (Fig. 5c, fourth lanes). We further observed that the major somatic linker histone H1.2 not to be associated with H1t-ChIP elute fraction, highlighting that the H1t-antibodies did not pull down H1.2 and its associated proteins, but were specific to the linker histone variant H1t (Fig. 5c, α-H1.2, third lane). Mass spectrometric analysis of H1t-associated protein complexes reflects the average picture at all the repeat-element chromatin domains. Repeat-class specific complexes could interact to modulate loci-specific functions in pachytene spermatocytes. In summary, these observations demonstrate the association of linker histone H1t with repressed repeat-chromatin domains in pachytene spermatocytes.

## Discussion

Recently, tremendous progress has been made towards understanding the molecular functions of linker histone H1 and its subtypes in developmental processes beyond their architectural roles in chromatin. The lack of studies on H1 function can be largely attributed to technical difficulties during the use of mass spectrometry approaches yielding reduced protein coverage and also the generation of subtype-specific antibodies. Raising variant-specific antibodies is a challenge. In this background, we have successfully generated H1t-specific antibodies in rabbit and validated its specificity by multiple assays.

### Linker histone variant H1t and TE repression

An important observation made in the present study is the predominant localization of linker histone variant H1t at repeat elements belonging to LINE and LTR. Even though repeat-element RNAs orchestrate gene expression patterns during early embryonic development [48, 49], paradoxically in the context of germ cells, the repeat elements need to be repressed to prevent mutagenesis and genome instability. Importantly, the expression of proteins like Dnmt3A, the enzyme that is responsible of DNA methylation of repeat elements, does not occur in spermatocytes, establishing methylation patterns in prespermatogonia become necessary in spermatocytes, because the defects are inherited during the pachytene interval. The loss of Dnmt3l leads to the upregulation of LINE and LTR transcripts in spermatogonia and spermatocytes, suggesting its role in TE repression phenomenon in the premeiotic germ cells. These epigenetic processes involving TE repression are important especially during pachytene interval, where scanning and apoptotic checkpoint mechanisms are in place. Importantly, defects in DNA methylation at TE elements results in shifting of recombination hotspots to non-canonical genomic sites, ultimately resulting in male infertility [50]. Based on the observation of the H1t-occupied repeat elements coinciding with occupancy of methylated CpGs, it is tempting to speculate that these repeat elements represent repressed chromatin domains in vivo. H1 has also been shown to contribute to the establishment of DNA methylation patterns in mESCs [51]; this provides additional support for the importance of H1t and its association with DNA methylation-associated genomic regions in pachytene spermatocytes. In *Arabidopsis thaliana*, linker histone H1 and DNA methylation jointly regulate gene expression patterns by repressing TE in vivo [52]. Thus, it is very likely that even in the context of mammalian spermatocytes, H1t and methylated CpGs might recruit specialized effector proteins to jointly repress TE chromatin domains (6A, 6B).

**Fig. 6:**
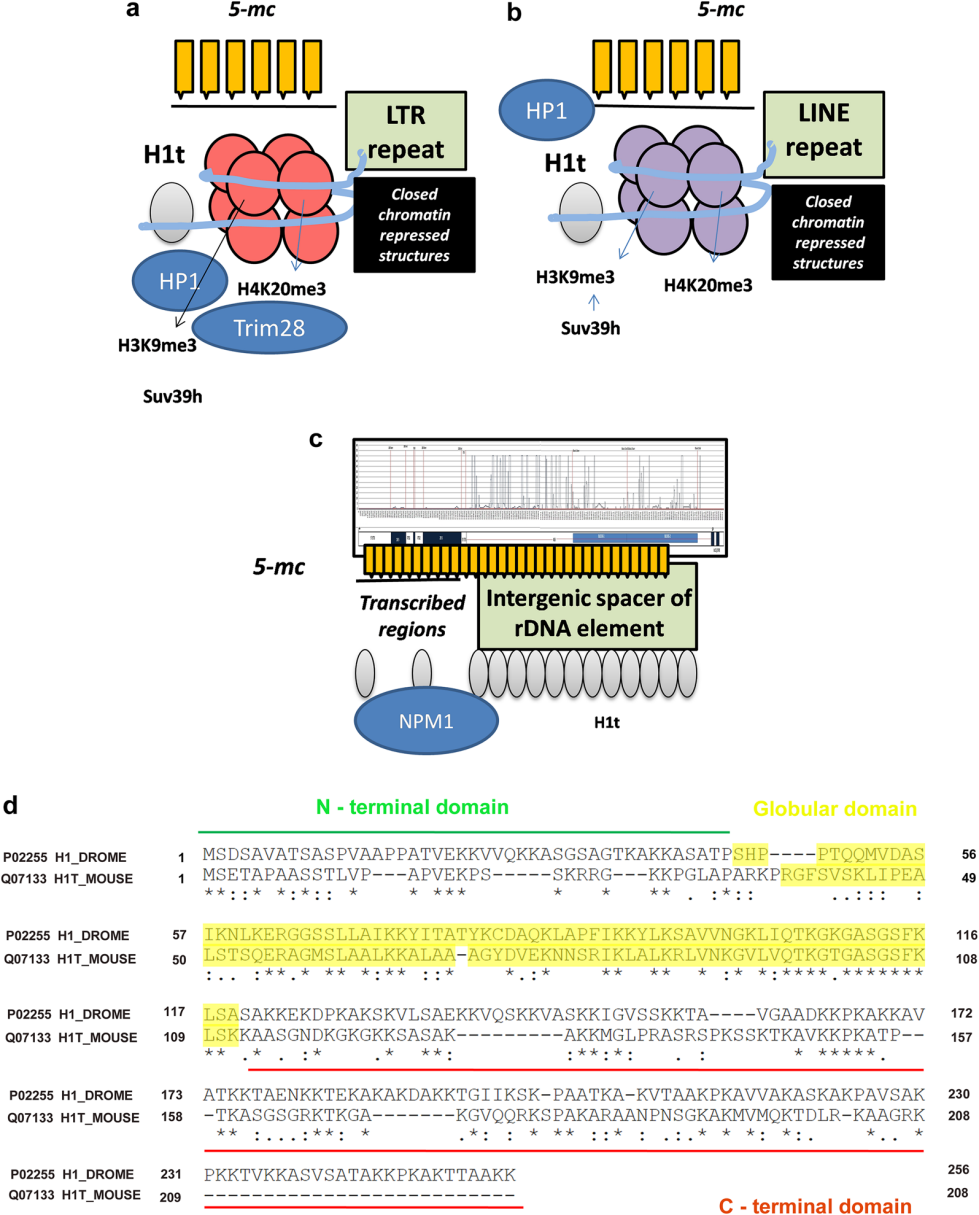
Model of H1t-containing chromatin domains in pachytene spermatocytes. **a**, **b** Extranucleolar localization of linker histone variant H1t is related to **a** LINE and **b** LTR repeat-element chromatin domains. This forms a chromatin template for binding of PIWI proteins, DNA methylation machinery (forming methylated CpGs), repressive histone modifications like H3K9me3 and H4K20me3 in pachytene spermatocytes. H3K9me3 nucleosomes are binding sites for HP1 proteins to mediate heterochromatinization at these target loci. **c** Predominant association of linker histone variant with the intergenic spacer of the rDNA element. H1t co-associates with methylated CpGs and nucleolar proteins like Npm1 at the rDNA element in pachytene-enriched P20 mouse testicular cells. **d** Alignment of protein sequences of mouse H1t with Drosophila H1. Both these proteins lack DNA-binding motifs like S/TPKK in its C-terminal domain

An interesting question that arises here is what determines the localization of H1t to these specific genomic loci. We observed a combination of 12 motifs to be significant in the total H1t peaks as identified through motif analysis using MEME (Additional file 2: Figure S2B). Whether the motif information of the target DNA sequence is sufficient for H1t binding in the pachytene spermatocyte genome remains to be further examined. Since we observed that the occupancy of the repeat elements is characteristic of linker histone H1t in pachytene spermatocytes, it could be possible that the unique C-terminus of H1t carry information to localize at repetitive elements. It is also possible that adaptor proteins might recruit histone H1t to these specific genomic loci. In this context, we would like to mention that PIWI proteins recruit H1 to TE loci to repress these chromatin domains in ovarian somatic cells (OSC) of Drosophila [53]. Therefore, proteins belonging to the PIWI pathway might regulate H1t localization at repeat elements in pachytene spermatocytes. This presumption is additionally supported by the fact that various proteins belonging to PIWI pathway like PIWIL1, Hspa2, Uhrf1, Trim28 are associated with H1t-containing chromatin domains. It is also known that C-terminal domain of H1 is required for recruitment of Piwi and TE silencing in Drosophila [53]. We were surprised to see that the Drosophila H1 and H1t protein sequences are similar in the C-terminal regions being devoid of various DNA-binding motifs like S/TPKK (Fig. 6d).

H1t-containing chromatin domains are also associated with repressive histone modifications like H3K9me3 and H4K20me3 in the spermatocyte genome. It will be interesting to decipher whether the association of H1t with methylated CpGs in the repeat elements is dependent on the piRNA–PIWI pathway or not. Despite ongoing efforts to characterize in-detail the mechanisms of TE repression in germ cells, the crosstalk between epigenetic pathways involving repressive histone modifications, DNA methylation, and piRNAs/PIWI proteins have not been delineated in great detail. We suspect H1t along with repressive histone H4K20me3 and H3K9me3 might set the template for TE repression in germ cells (Fig. 6a, b). Crosstalk between H1t and repressive histone modifications could occur; working with conjunction with piRNA–PIWI pathway and heterochromatin proteins can lead to formation of repressed chromatin structures in vivo.

Biochemical and biophysical studies have shown that linker histone H1t, due to lack of S/TPKK motif in its C-terminus, is a poor condenser of chromatin and also generates a relaxed chromatin structure with oligonucleosomal template in vitro [19–24]. Based on this propensity, we would like to propose that H1t-mediated relaxed chromatin structure at the repeat element chromatin domains would facilitate the initiation of recruitment of repression machinery, resulting in heterochromatin formation at these sites.

### H1t and nucleolus

As explained earlier, H1t was previously shown to be localized to the nucleolus in mouse spermatocytes and human cancer cell lines [31]. We could confirm their observations even in spermatocytes. Further evidence for association of linker histone H1t with nucleolar chromatin comes from our observations that we could pick up nucleolar proteins such as Npm1, Numa1, Ncl in the H1t IP chromatin by mass spectrometry. An interesting observation made in the present study is the predominant association of H1t peaks with the intergenic spacer of the rDNA element. There have been numerous reports of the role of intergenic spacer in modulating rDNA transcription [54, 55]. macroH2A has been shown to repress rDNA transcription in human HeLa and mouse ES cells [56]. Since we observed macroH2A be associated with H1t-ChIP fraction as determined by mass spectrometry, this suggests that H1t could function with macroH2A to modulate rDNA transcription in germ cells, providing further support to the possible role of H1t in rDNA transcriptional dynamics. The rDNA element is a host to repetitive elements of various classes, SINE being the predominant class [43].

Both the nucleolar and extra-nucleolar localization of H1t occurs in the repeat regions of the mouse pachytene genome. In mammalian cells, chromatin is organized into structural and functional compartments like TADs (topologically associated domains), LADs (lamin-associated domains), etc. [57–62]. Recently, nucleolus-associated domains (NADs) have been discovered in HeLa cervical carcinoma and HT1080 fibrosarcoma cells [63, 64]. Various repeat elements like LTR are enriched in NADs and inter NADs [65]. It would be interesting to see whether this kind of nucleolar organization exists in pachytene spermatocytes, if yes, then does H1t influence structure of NADs and inter NADs via its association with repeat elements.

Nucleosomal retention occurs in repetitive sequences associated with intergenic and intronic regions in mammalian sperm [66, 67]. This epigenetic landscape sets up an important marker for paternally derived nucleosomes in preimplantation embryos. Since H1t has been demonstrated to occur at repeat sequences associated with intergenic and intronic regions, this begs for the question of which linker histone variant(s) or PTMs mark these functionally important genomic regions in the mature sperm. An important point to be noted here is that H1t expression is restricted till early round spermatids in mouse. Other variants could replace H1t at repeat elements during spermiogenesis. One candidate variant, HILS1 is also enriched at the LINE1 elements in rat spermatids [68]. Also, in vitro studies have demonstrated that H1t can be poly ADP-ribosylated (PAR), and PAR-modified H1t promotes chromatin compaction [69]. Disturbance in PAR metabolism causes retention of H1t, HILS1 and core histones in the mature spermatozoan [70]. Recently, various post-translational modifications have been characterized on H1t obtained from spermatocyte and round spermatocyte cell populations by mass spectrometry [71]. It would also be interesting to see whether PTMs on H1t mark specific repeat element subclasses. It will also be exciting to determine the loci-specific functions of H1t PTMs in repeat repression phenomena. Specific marking of chromatin territories might occur in various stages of germ cell development, wherein different histone variants with their PTMs might contribute to unique functions in shaping the epigenetic landscape important for male fertility and transgenerational inheritance. An intriguing question that arises at this juncture is why histone H1t knockout mice do not show any defect in fertility. It is worth mentioning here that the histone TH2B knockout mice are also fertile, but in these mice, the somatic histones H2B, H3 and H4 acquire compensatory histone PTMs that could substitute the role of TH2B in spermatogenesis [72]. Thus, a more detailed investigation of chromatin modifications in histone H1t-deficient mice is necessary to understand the biological function and relevance of histone H1t in mammalian spermatogenesis.

## Conclusions

Very few studies have shed light on the biological roles of testicular linker histone variants in the context of differentiation of male germ cells. The present study is focussed towards understanding the genomic and chromatin features of H1t-occupied chromatin domains. We observe that linker histone H1t is not closely associated with TSS, active gene promoters, DSB hotspots and open chromatin regions. We have extensively characterized the nucleolar and extranucleolar localization features of linker histone variant H1t and found that H1t to be predominantly associated with repeat element chromatin domains in pachytene-enriched P20 mouse testicular cells. These chromatin domains are positive for methylated CpGs, repressive histone modifications like H3K9me3 and H4K20me3, protein marks like piRNA–PIWI pathway components, heterochromatin proteins, etc. We propose that H1t might induce local chromatin relaxation to recruit proteins effectors necessary for repression of these repeat elements.

## Materials and methods

### Cloning and expression of C-terminal protein fragment of H1t

The coding sequence (CDS) corresponding to 112–207 amino acid residues of H1t protein was cloned using specific primers (forward primer-ATAGAAGCTTGCAGCTTCAGGCAACGAC and reverse primer-ATATGCGGCCGCCTTTCTTCCTGCTGCCTTCC) into pET22b(+) vector using HindIII and NotI restriction sites. The expression vector was transformed in Rosetta strain of *E. coli*, and His-tagged proteins were purified using Ni–NTA purification method.

### Antibody generation

The recombinant protein (C-terminal fragment of H1t) was injected into rabbits, and the 14-day cycle of antibody generation was followed. Immunoglobulins were purified by caprylic acid-based purification method. Antigen-affinity-based purification with the Sulfolink columns containing immobilized proteins was used to purify the H1t-specific antibodies. The H1t antibody was outsourced from the Abgenex company (Bhubaneshwar, India).

### ELISA

The recombinant proteins were used at 200 ng per well. The pre-bleed and immune sera were used at 1:5000 dilution. Goat anti-rabbit HRP was used as the secondary antibody at 1:5000 dilution. TMB (3,3′,5,5′-tetramethylbenzidine) was used as the substrate for color development. After 3 min of enzyme–substrate reaction, the plate was read at 450 nm.

### Extraction of linker histones from rodent testes

The linker histones along with other acid soluble nuclear proteins were extracted from rat or mouse testes by perchloric acid method [73, 74]. Briefly, the purified nuclei were resuspended in 10% perchloric acid, homogenized and kept on ice for 30 min. The sample was centrifuged for 10,000*g* for 10 min at 4 °C to pellet the residual chromatin. The proteins were extracted from the supernatant using 30% trichloroacetic acid (TCA). After incubation on ice for 30 min, the proteins were recovered by centrifugation at 12000*g* at 4 °C for 10 min. The protein pellets were sequentially washed once with cold acetone containing 0.05% HCl and twice with ice-cold acetone. The pellet obtained was dried, dissolved in water and stored in aliquots at − 20 °C.

### Extraction of histones from rodent testes

Histones were extracted from the rodent testes using the published protocol [75]. Briefly, the cell pellet was resuspended in hypotonic lysis buffer (10 mM Tris–Cl pH 8.0, 1 mM KCl, 1.5 mM MgCl_2_ and 1 mM DTT, 1× protease inhibitor cocktail) and then incubated on rotator for 30 min at 4 °C. The nuclei were pelleted by centrifugation at 10,000*g*, 10 min, 4 °C. The pellet was resuspended in 0.4 N H_2_SO_4_, incubated on rotator for 30 min at 4 °C. Histones were then precipitated using 33% TCA (final concentration) and the histone pellet was then washed with ice-cold acetone two times before resuspending in appropriate volume of water.

### Preparation of meiotic spreads from testicular cells

Meiotic spreads were prepared according to the published protocol [76].

### ChIP-sequencing of linker histone variant H1t in P20 mouse testicular cells

Chromatin immunoprecipitation (ChIP) was carried out using the published protocol [77]. Briefly, P20 mice testes were dissected in 1× PBS (with 1% formaldehyde), incubated for 10 min on a rotating wheel at room temperature. Quenching to remove formaldehyde was done using glycine (250 mM final concentration). The pellet was washed with 1× PBS multiple times. The suspension was filtered using a 40 µm filter and then centrifuged at 500*g* for 5 min at 4 °C. The pellet was resuspended in Buffer A (10 mM Tris pH 8.0; 10 mM KCL; 0.25% Triton-X-100; 1 mM EDTA; 0.5 mM EGTA; 1× protease inhibitor cocktail from Roche), and incubated on ice for 5 min. Again, the contents were centrifuged at 500*g* for 5 min at 4 °C. The pellet was then resuspended in Buffer B (10 mM Tris pH 8.0; 200 mM NaCl; 1 mM EDTA, 0.5 mM EGTA; 1× protease inhibitor cocktail), incubated on ice for 10 min, centrifuged again at 500*g* for 5 min at 4 °C. Finally, the pellet was resuspended in SDS lysis buffer (1% SDS, 10 mM EDTA, 50 mM Tris pH 8.0, 1× protease inhibitor cocktail). The contents were incubated on a rotating wheel for 30 min at 4 °C. Sonication was carried out 40 cycles, and chromatin fractions were diluted 1/10 times with the dilution buffer (5 mM Tris PH 8.0, 140 mM NaCl, 0.5% Triton-X-100, 0.05% sodium deoxycholate, 0.5 mM EGTA) before subjecting it to antibody binding overnight at 4 °C on a rotating wheel. Dynabeads were added the next day, washes were given with W1 (10 mM Tris pH 8.0, 150 mM KCl, 0.50% NP-40, 1 mM EDTA), W2 (10 mM Tris pH 8.0, 100 mM NaCl, 0.10% sodium deoxycholate, 0.50% Triton-X-100), W3a (10 mM Tris pH 8.0, 400 mM NaCl, 0.10% sodium deoxycholate, 0.50% Triton-X-100), W3b (10 mM Tris pH 8.0, 500 mM NaCl, 0.10% sodium deoxycholate, 0.50% Triton-X-100), W4 (10 mM Tris pH 8.0, 250 mM LiCl, 0.50% sodium deoxycholate, 0.50% NP-40, 1 mM EDTA), W5 (10 mM Tris pH 8.0, 1 mM EDTA) wash buffers. The complexes were eluted from input, rabbit IgG control ChIP, and H1t ChIP samples using elution buffer (50 mM Tris pH 8.0, 1% SDS, 1 mM EDTA) and then incubated at 65 °C overnight with intermittent shaking. The supernatants recovered from the tubes were subjected to RNase A and proteinase K digestions at 37 °C. DNA was then extracted from the elute fractions using the phenol–chloroform method. The input and ChIP DNA libraries were prepared using the NEBNext Ultra II DNA library preparation kit. The resulting libraries were quantified before getting sequenced on the Illumina HiSeqX system to generate 2X150 bp sequence reads.

### Computational data analysis

All the four samples (input controls two and H1t ChIP two samples—the raw Illumina sequenced files in FASTQ) were quality checked with FastQC (version 0.11.5). Each sample (two input controls and two H1t ChIP files) were then aligned to mouse genome version (GRCm38.p5) using Bowtie2 [78] with alignment parameters: -D 15 -R 2 -N 1 -i S,1,0.75 –local. The aligned samples (SAM/BAM files) were used to find the peaks with MACS2 version 2.1.2, with parameters: #effective genome size = 1.87e+09, #band width = 300 #model fold = [5, 50], #pvalue cutoff for narrow/strong regions = 5.00e–02, #pvalue cutoff for broad/weak regions = 5.00e−02, #Range for calculating regional lambda is: 1000 bp and 10000 bp, #Broad region calling is on, #Paired-End mode is on. The ChIP-sequencing dataset is given in Additional file 6. Annotation of H1t-bound genomic regions was carried out using HOMER (Additional file 7). The read concentration of H1t peaks was compared against the already published datasets of active gene promoters (GSE93955), TSS (GENCODE), DSB hotspots (GSE93955) and ATAC-sequencing data (GSE102954) and plotted using ngsplot.r tool [34].

Various in-house Perl scripts and database comparison scripts at BioCOS Life Sciences were used to do the rest of the analysis like rDNA, Motif, and comparative methylation, etc. FASTA sequences corresponding to the rDNA element were obtained from NCBI [43]. A FASTQ file was generated from the significant peaks obtained from the MACS analysis to enable the alignment of the peaks on to rDNA FASTA sequences. After the peaks were aligned to the rDNA element using Bowtie using the rDNA FASTA as reference file, the tag densities were then computed on the various regions of the rDNA element (Fig. 4a).

The raw bisulfite sequencing data for P20 mouse testicular cells were obtained from the Sequence Read Archive (SRA) database (SRS557654). SRR1170580 is the Single end WT sample from SRS557654, is used for extraction of methylation locations and compare locations with H1t peaks. The reads were aligned using Bowtie2 onto the reference genome of mouse (GRCm38.p5)., The methylation analysis was done using bismark_v0.22.1 [79] and the methylated markers were isolated. (./bismark_v0.22.1/bismark –parallel 4 –bowtie2 -D 5 -R 1 -N 0 -L 22 –local -un -o AlignmentMeth_SE_SRS557654 –prefix AlignMethyl Bismark Genome mm10/single_end../Raw_SE_sample_SRS557654/SRR1170580.fastq.bz2). The methylated markers were isolated (./bismark_v0.22.1/bismark_methylation_extractor -s –comprehensive -o Methylated_Regions/–cytosine_report –bedGraph –parallel 2 AlignmentMeth_SE_SRS557654/AlignMethylBismark.SRR1170580_bismark_bt2.bam –genome_folder Genome_mm10/). Then the overlapping of the methylated markers (bases) of the database (SRS557654) with the H1t ChIP-seq Peaks was carried out using in-house perl scripts and R (https://www.r-project.org/) (Fig. 4b).

Motif analysis was carried out using MEME software [80]. The parameters set for MEME- Search-size → 100,000, DataBase: JASPAR/JASPAR2018_CORE_non-redundant.meme,-ccut(cut sequence from center): 0, -meme-searchsize: 100,000,-filter-thresh: 0.0001(e Value),-meme-minw: 6,-meme-maxw: 50,-meme-p: 6,-meme,nmotifs: 15,-dreme-e: 0.0001,-dreme-m: 15,-centrimo-ethresh: 0.0001.

### Mass spectrometric identification of proteins associated with H1t-containing chromatin fragments

Immunoprecipitation of H1t-associated protein complexes was carried out, and the proteins were extracted from the beads using the elution buffer of the Pierce co-IP kit. The eluted proteins were resolved on 15% SDS gel and the gel was subjected to Coomassie staining. The stained wells corresponding to the IgG and IP lanes were outsourced for mass spectrometry to identify the interacting proteins. The detailed experimental protocol of the mass spectrometry was followed according to the published protocol [81]. The list of H1t-associated proteins is given in Additional file 8. The comprehensive list of common proteins between H1t and the heterochromatin set [46] is given in Additional file 9.

### Western blotting

The proteins were resolved by gel electrophoresis and transferred onto the nitrocellulose membrane. The blots were blocked using blocking solution (5% skim milk in 1× TBS). The primary antibodies were made in 1% skim milk (prepared in 1× TBS) and added onto the blots and were left for overnight incubation in shaking conditions at 4 °C. Next day, two washes were given using 0.1% TBST (0.1% Tween-20 in 1× TBS) for 5 min each. The secondary antibodies prepared in 1% skim milk was added to the blots and left for shaking for 1-h incubation at room temperature. 2–3 washes were given with 0.1% TBST before developing the blot using Millipore Immobilon Forte Western HRP Substrate kit (Cat number-WBLUF0100) in the Biorad Chemidoc Touch imaging system.

For stripping of proteins from the blot, 1X Millipore Reblot Plus Strong Antibody Stripping Solution (Cat number-2504) was added to the blot and left for shaking at room temperature for 20–25 min. The blot was developed using the ECL kit to check for any signal on the membrane. Two washes were then given with blocking solution (5% skim milk in 1× TBS) for 5 min each and then proceeded with incubation with the primary antibody solution.

### Protein competition assay

For the protein competition assays, H1t or H1.2 antibodies were preincubated with 10 µg of H1t C-terminal protein fragment before their addition to the blot.

**Table:** List of antibodies used in the present study

Reagents				
Antibodies	Host	Company name	Cat number	Application
Scp3	Mouse	Abcam	ab97672	IF, WB
HP1β	Mouse	Millipore	MAB3448	WB
HP1γ	Mouse	Millipore	05-690	WB
Trim28 (Kap1)	Mouse	Abcam	ab22553	WB
Hormad1	Rabbit	Abcam	ab155176	WB
NPM1	Mouse	Abcam	ab10530	WB
Hspa2	Rabbit	Invitrogen	PA5-81895	WB
PIWIL1	Rabbit	Invitrogen	PA5-21051	WB
H3K9me3	Rabbit	Millipore	07-442	WB, ChIP
H4K20me3	Rabbit	Abcam	ab9053	WB, ChIP
H1.2	Rabbit	Abcam	ab181973	WB
H3K4me3	Mouse	Abcam	ab12209	WB, ChIP

*WB* western blotting, *ChIP* chromatin immunoprecipitation, *IF* immunofluorescence

## Supplementary information

**Additional file 1: Figure S1. A**. Coomassie-stained gel showing the successful purification of His tagged C-terminal fragment of H1t. The purity of proteins was determined after elution using 100 mM, 200 mM, 300 mM, and 400 mM imidazole, wherein the protein fractions were obtained after elution using 300/400 mM concentration of imidazole. (**B**-**C)** Validation of specificity of the H1t antibody towards the recombinant H1t C-terminal protein fragment by ELISA using B. Immune sera and C. Purified antibody. The sera, as well as purified antibodies, showed reactivity against the H1t C-terminal protein fragment. The color code schemes have been indicated on the right of the figures. **D**. Immunoblotting using anti-H1t and anti-H1.2 antibodies probing against acid extracted histones from liver of 20-day old mouse. H1t is absent in the liver acid extracts, whereas the somatic H1.2 is found in the liver histone extracts. The ponceau stained blots and coomassie blue-stained SDS gel are given for reference.

**Additional file 2: Figure S2. A**. Western blotting analysis of rat testicular perchloric acid extracts using H1t and H1.2 antibodies confirming the specificity of the H1t and H1.2 antibodies. The blots to the right are the immunoblotting results obtained after preincubation of the H1t and H1.2 antibodies with the recombinant H1t C-terminal antigen. **B**. Immunoblotting performed with H1t and H1.2 antibodies probed against rat testicular acid extracts. The blots to the left represent the immunoblotting pattern obtained against the rat testicular acid extracts. The blots to the right indicate the results obtained after performing the protein competition assay with the H1t C-terminal antigen. The reactivity of the H1t antibodies but not H1.2, was abolished upon preincubation with the recombinant H1t C-terminal protein fragment. Ponceau stained blots and Coomassie-stained gel are given for reference.

**Additional file 3: Figure S3. A**. Immunostaining pattern of linker histone variant H1t across various stages of meiotic prophase I. Staining of anti-H1t and anti-Scp3 across leptotene (L, first panel), leptotene-zygotene (L/Z, second panel), zygotene (Z, third panel), and pachytene (P, fourth and fifth panels). **B**. Profile of DNA fragments obtained after 10, 20, 30, 35, and 40 cycles of sonication of P20 mouse testicular chromatin. 100-300 bp of fragment sizes were predominantly obtained after 40 cycles of sonication were used further for ChIP assays. Linker histone variant H1t is not associated with histone mark H3K4me3-containing chromatin domains- **C**. IP was carried out using the anti-H3K4me3 antibody where the H3K4me3 and H1t were probed by western blotting. **D**. Reciprocal IP using the anti-H1t antibody where H3K4me3 and H1t were detected by western blotting. The antibodies used for the western blotting are indicated in alpha alongside the blot. Ponceau stained blots are given for reference.

**Additional file 4: Figure S4. A**. Peak to peak comparison of H1t ChIP-sequencing peaks with DSB hotspots, total H3K4me3 marks, Dmc1, TSS-associated H3K4me3, Hotspot-associated H3K4me3, PRDM9 and ATAC sequencing datasets. 99% of the H1t peaks overlap with methylated CpGs in the rDNA element. The y-axis represents the number of methylated H1t peaks weighted by the number of methylated bases, and the x-axis represents the individual H1t peaks that are aligned on the rDNA element. The various regions of the rDNA element have been labelled below the peak distribution maps.

**Additional file 5: Figure S5. A**. Table showing the detailed comparison of H1t peaks and methylated CpGs in the extranucleolar (non rDNA) and nucleolar (rDNA) regions of the mouse genome. **B**. Venn Diagram showing the distribution of methylated H1t peaks in the rDNA and the extranucleolar regions of the mouse genome. **C**. Table of motifs identified of H1t bound genomic regions in pachytene spermatocytes using MEME software.

**Additional file 6.** ChIP-sequencing peaks of H1t in P20 mouse testicular cells.

**Additional file 7.** Annotation of H1t peaks using HOMER.

**Additional file 8.** H1t-associated proteins obtained after mass spectrometry.

**Additional file 9.** H1t and associated heterochromatin-related proteins.

## Data Availability

The ChIP-sequencing dataset containing the raw and processed files are deposited in Gene Expression Omnibus (GEO) (GSE142081).

## Abbreviations

DDR: DNA double-strand break repair
TSS: Transcription start sites
LTR: Long terminal repeats
LINE: Long interspersed nuclear element
SINE: Short interspersed nuclear element
TE: Transposable element
rDNA: Ribosomal DNA
ChIP: Chromatin immunoprecipitation
ATAC-seq: Assay for Transposase-Accessible Chromatin using sequencing
piRNA: PIWI-interacting RNA
PIWI: P-element induced wimpy testis
