# Supplementary material for: Linker histone variant H1t is closely associated with repressed repeat-element chromatin domains in pachytene spermatocytes
Source: Epigenetics Chromatin. 2020 Mar 4;13:9. doi: 10.1186/s13072-020-00335-x (PMC7057672; doi:10.1186/s13072-020-00335-x)

Additional file 1: Figure S1

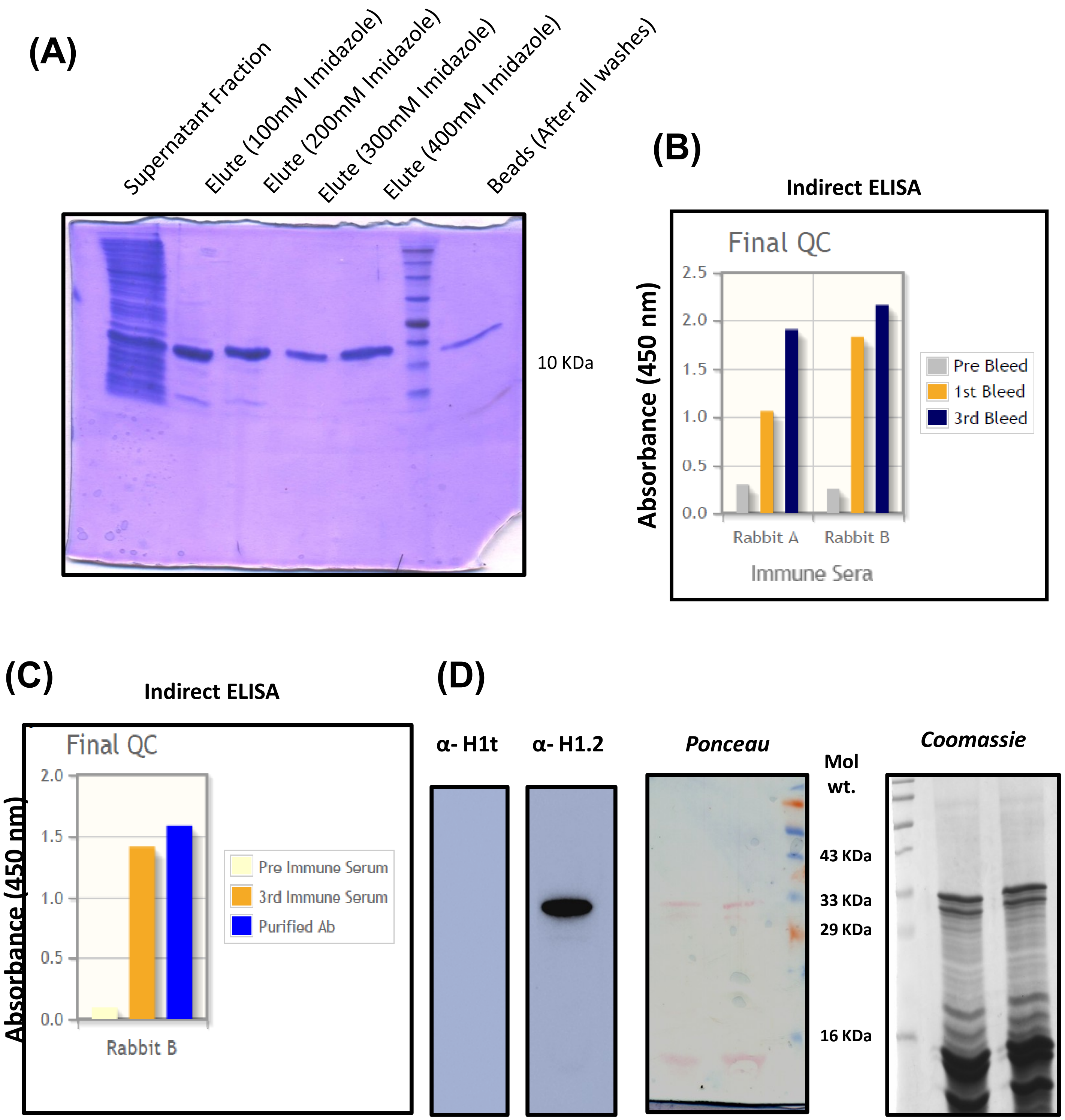

Supplement: Supplementary file 1 — Additional file 1: Figure S1. A. Coomassie-stained gel showing the successful purification of His tagged C-terminal fragment of H1t. The purity of proteins was determined after elution using 100 mM, 200 mM, 300 mM, and 400 mM imidazole, wherein the protein fractions were obtained after elution using 300/400 mM concentration of imidazole. (B-C) Validation of specificity of the H1t antibody towards the recombinant H1t C-terminal protein fragment by ELISA using B. Immune sera and C. Purified antibody. The sera, as well as purified antibodies, showed reactivity against the H1t C-terminal protein fragment. The color code schemes have been indicated on the right of the figures. D. Immunoblotting using anti-H1t and anti-H1.2 antibodies probing against acid extracted histones from liver of 20-day old mouse. H1t is absent in the liver acid extracts, whereas the somatic H1.2 is found in the liver histone extracts. The ponceau stained blots and coomassie blue-stained SDS gel are given for reference. [file 13072_2020_335_MOESM1_ESM.pdf]
