# Supplementary figures and images for: Linker histone variant H1t is closely associated with repressed repeat-element chromatin domains in pachytene spermatocytes
Source: Epigenetics Chromatin. 2020 Mar 4;13:9. doi: 10.1186/s13072-020-00335-x (PMC7057672; doi:10.1186/s13072-020-00335-x)

# Additional file 2: Figure S2

(A)

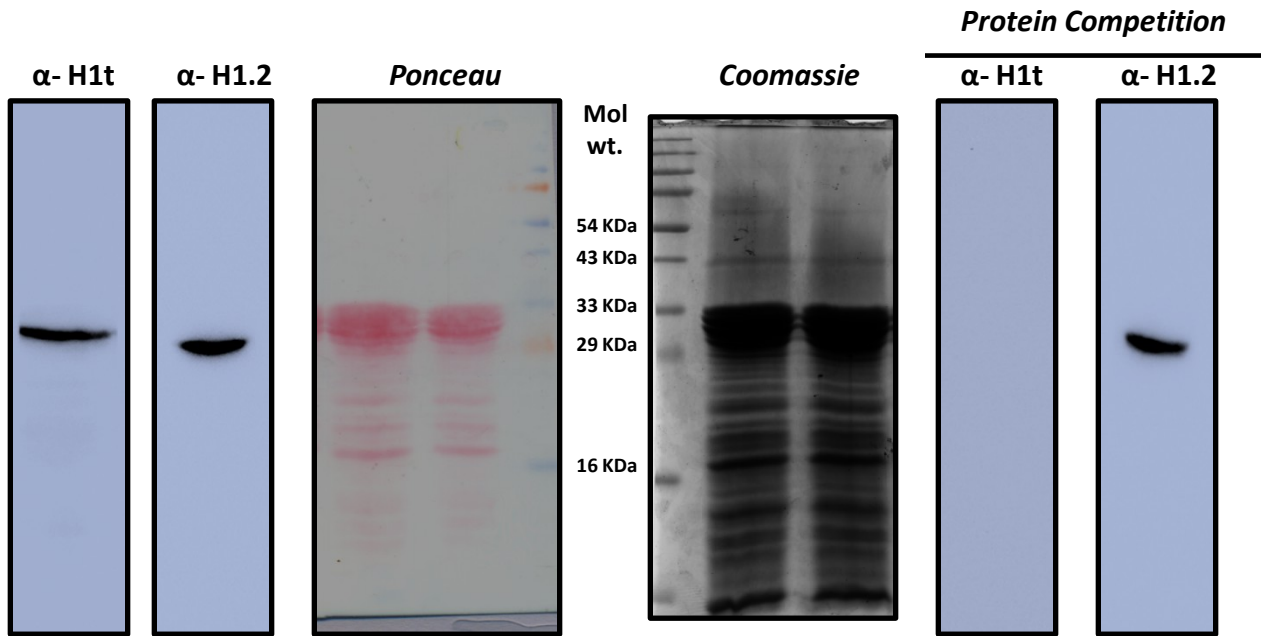

(B)

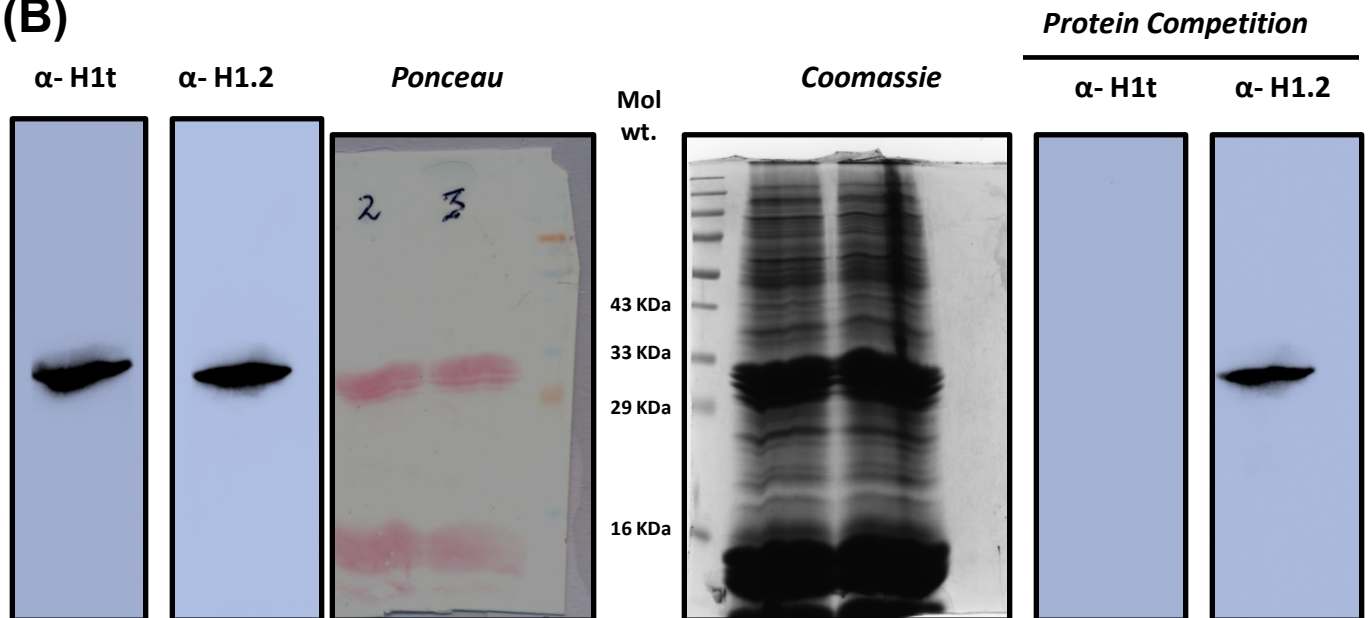

Supplement: Supplementary file 2 — Additional file 2: Figure S2. A. Western blotting analysis of rat testicular perchloric acid extracts using H1t and H1.2 antibodies confirming the specificity of the H1t and H1.2 antibodies. The blots to the right are the immunoblotting results obtained after preincubation of the H1t and H1.2 antibodies with the recombinant H1t C-terminal antigen. B. Immunoblotting performed with H1t and H1.2 antibodies probed against rat testicular acid extracts. The blots to the left represent the immunoblotting pattern obtained against the rat testicular acid extracts. The blots to the right indicate the results obtained after performing the protein competition assay with the H1t C-terminal antigen. The reactivity of the H1t antibodies but not H1.2, was abolished upon preincubation with the recombinant H1t C-terminal protein fragment. Ponceau stained blots and Coomassie-stained gel are given for reference. [file 13072_2020_335_MOESM2_ESM.pdf]

# Additional file 3: Figure S3

(A)

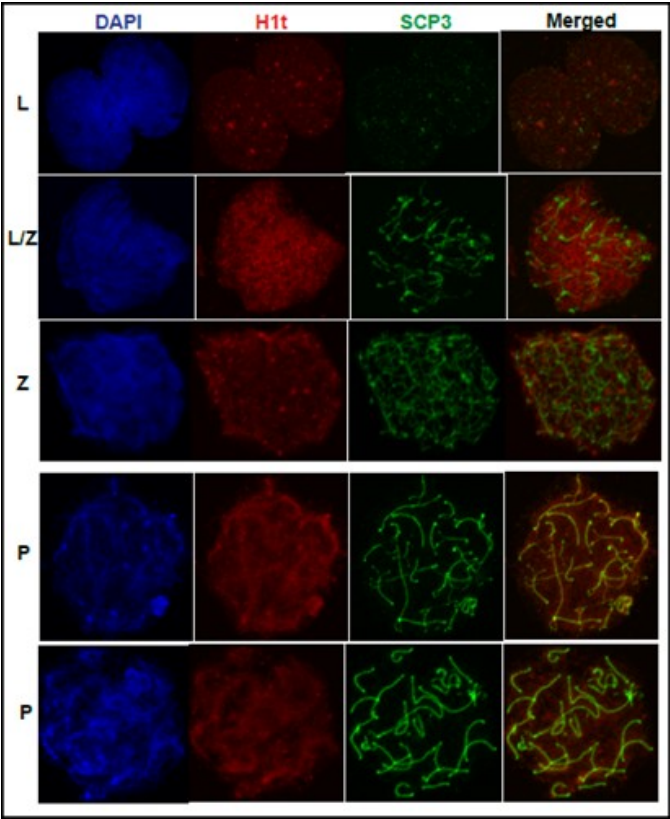

(B)

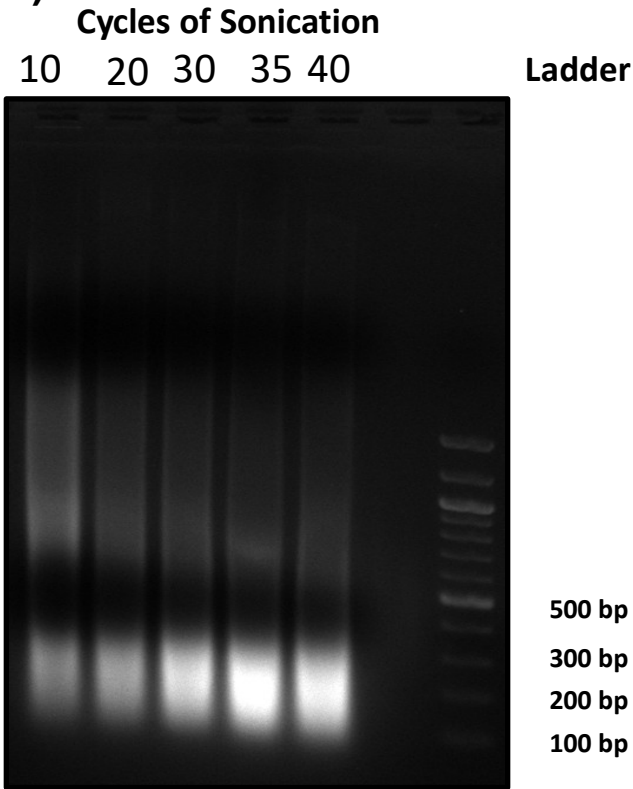

(C)

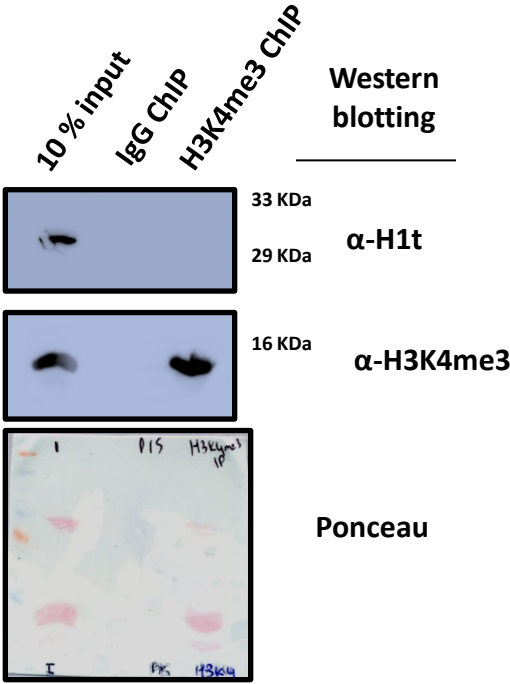

(D)

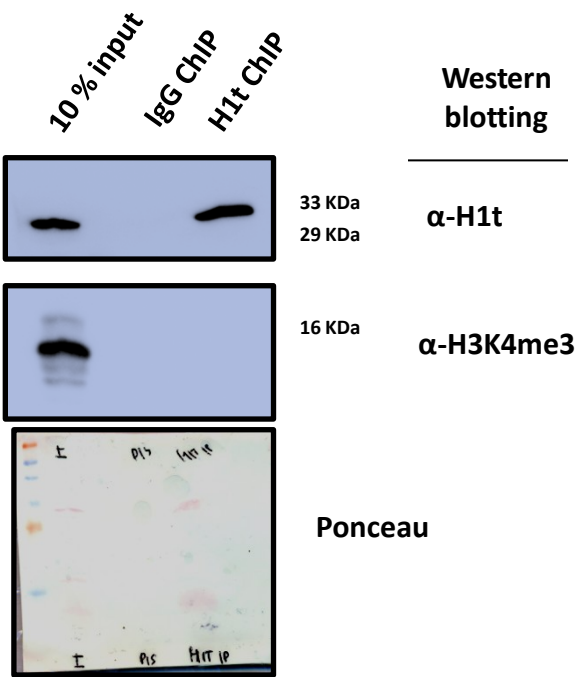

Supplement: Supplementary file 3 — Additional file 3: Figure S3. A. Immunostaining pattern of linker histone variant H1t across various stages of meiotic prophase I. Staining of anti-H1t and anti-Scp3 across leptotene (L, first panel), leptotene-zygotene (L/Z, second panel), zygotene (Z, third panel), and pachytene (P, fourth and fifth panels). B. Profile of DNA fragments obtained after 10, 20, 30, 35, and 40 cycles of sonication of P20 mouse testicular chromatin. 100-300 bp of fragment sizes were predominantly obtained after 40 cycles of sonication were used further for ChIP assays. Linker histone variant H1t is not associated with histone mark H3K4me3-containing chromatin domains- C. IP was carried out using the anti-H3K4me3 antibody where the H3K4me3 and H1t were probed by western blotting. D. Reciprocal IP using the anti-H1t antibody where H3K4me3 and H1t were detected by western blotting. The antibodies used for the western blotting are indicated in alpha alongside the blot. Ponceau stained blots are given for reference. [file 13072_2020_335_MOESM3_ESM.pdf]
