# Supplementary material for: Linker histone variant H1t is closely associated with repressed repeat-element chromatin domains in pachytene spermatocytes
Source: Epigenetics Chromatin. 2020 Mar 4;13:9. doi: 10.1186/s13072-020-00335-x (PMC7057672; doi:10.1186/s13072-020-00335-x)

Additional file 4: Figure S4

(A)

Data Source	Total Number of peaks	Peak Overlap	Total H1t peaks	Percent Overlap (w.r.t H1t peaks)
GSE35498 B6 DSB hotspots	18303	865	48681	1.78 %
GSE35498 H3K4me3	89116	2372	48681	4.87 %
GSE93955 Dmc1	14768	633	48681	1.30 %
GSE93955 H3K4me3 Common (TSS)	41008	1164	48681	2.39 %
GSE93955 H3K4me3 B6 specific (DSB Hotspots)	10829	265	48681	0.54 %
GSE93955 PRDM9	2601	51	48681	0.10 %
GSM2751130 ATAC Seq	24755	304	48681	0.62 %

(B)

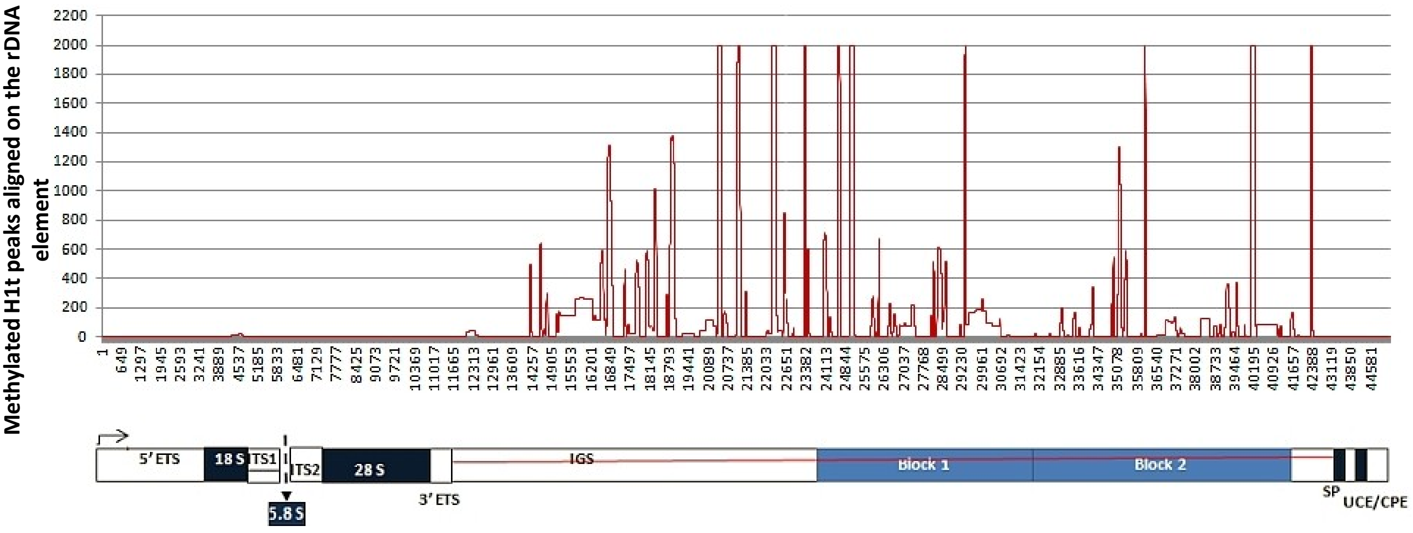

Supplement: Supplementary file 4 — Additional file 4: Figure S4. A. Peak to peak comparison of H1t ChIP-sequencing peaks with DSB hotspots, total H3K4me3 marks, Dmc1, TSS-associated H3K4me3, Hotspot-associated H3K4me3, PRDM9 and ATAC sequencing datasets. 99% of the H1t peaks overlap with methylated CpGs in the rDNA element. The y-axis represents the number of methylated H1t peaks weighted by the number of methylated bases, and the x-axis represents the individual H1t peaks that are aligned on the rDNA element. The various regions of the rDNA element have been labelled below the peak distribution maps. [file 13072_2020_335_MOESM4_ESM.pdf]
