# Supplementary material for: Linker histone variant H1t is closely associated with repressed repeat-element chromatin domains in pachytene spermatocytes
Source: Epigenetics Chromatin. 2020 Mar 4;13:9. doi: 10.1186/s13072-020-00335-x (PMC7057672; doi:10.1186/s13072-020-00335-x)
Supplement: Supplementary file 5 — Additional file 5: Figure S5. A. Table showing the detailed comparison of H1t peaks and methylated CpGs in the extranucleolar (non rDNA) and nucleolar (rDNA) regions of the mouse genome. B. Venn Diagram showing the distribution of methylated H1t peaks in the rDNA and the extranucleolar regions of the mouse genome. C. Table of motifs identified of H1t bound genomic regions in pachytene spermatocytes using MEME software. [file 13072_2020_335_MOESM5_ESM.pdf]

Additional file 5: Figure S5

(A)

Peak Type	Total number of peaks	Number of unique peaks (duplicates removed)
Total H1t peaks	48681	48681
Non rDNA and methylated peaks	38782	38782
Non Methylated	3961	3961
rDNA and methylated	12550	5938
Overall number of unique peaks		48681

(B)

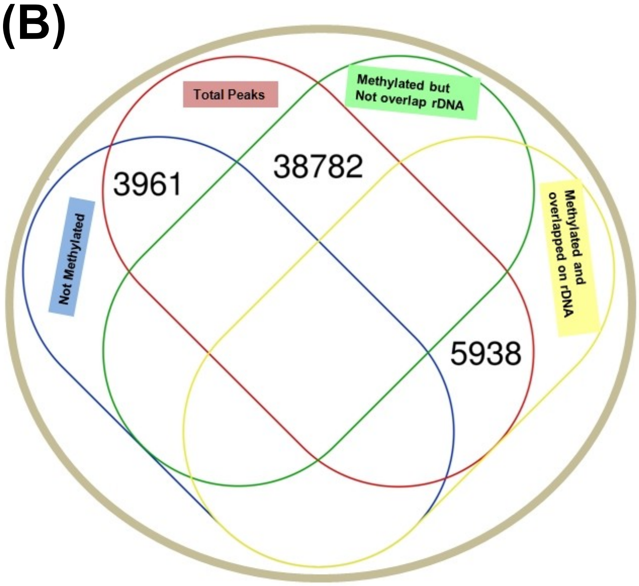

Methylation status of the enriched chip-seq peaks

(C)

Motif Serial no	MEME Motif ID	Motif Sequence	E value	Motif Width	Total Occurrences
Motif-1	MEME-8	ARGRRRRRRRRRRARRRRRRRARRVAGVM	3E-17	29	16607
Motif-2	MEME-6	TTTTTTTTTTTWWTTNTTTWTTTTTTTWT	3.9E-29	29	16215
Motif-3	MEME-9	SCBGGSMGTGGTGGCRCACGCCTTTAATCCCAGCACTYGGGAGGCAGAG G	4.5E-24	50	14623
Motif-4	MEME-10	CAAGGAGCTRAAGGGRTYTGCAACCCTATAGGWGGAACAAC	3.3E-10	41	13824
Motif-5	MEME-5	GCTGGAGAGATGGCTCAGYGGTTAAGAGC	1.9E-36	29	13553
Motif-6	MEME-4	TGAGTTCGAGGCCAGCCTGGTCTACARAGTGAGTTCC	1.4E-39	37	12466
Motif-7	MEME-11	ATGRGATCTGAYGCCCTCTTCTGGTGT	0.000000053	27	11117
Motif-8	MEME-3	ATGGTTGTGAGCCACCATGTGGTTGCTGG	2.2E-36	29	10716
Motif-9	MEME-12	GGGATCCATCCCATAWKCAGMCACCAAACCCAGACACTATTGCWKATG CC	0.000035	50	9593
Motif-10	MEME-7	TTGGCCCTGGCRTTCCCCTGTACTGGGGCATATAAAGTTTGCAAGWCCA A	1.1E-17	50	9481
Motif-11	MEME-1	CACACACACACACACACACACACACACAC	3.7E-52	29	4058
Motif-12	MEME-2	YTCTYTCTYTYTYTCTYTYTYTYTYTCTYTYTYTYTYTYTY	1.9E-71	41	3646
